# Supplementary material for: A community-driven resource for genomic epidemiology and antimicrobial resistance prediction of Neisseria gonorrhoeae at Pathogenwatch
Source: Genome Med. 2021 Apr 19;13:61. doi: 10.1186/s13073-021-00858-2 (PMC8054416; doi:10.1186/s13073-021-00858-2)
Supplement: Supplementary file 1 — Additional file 1: Table S1. List of studies included in the antimicrobial resistance benchmark analyses. Table S2. Point mutations and genes associated with antimicrobial resistance detected on the WHO 2016 reference panel. Table S3. Summary of the benchmark analysis. Table S4. List of genetic mechanisms detected on the test benchmark. Table S5. List of genetic mechanisms detected on the validation benchmark. Table S6. Public collections in N. gonorrhoeae Pathogenwatch. Table S7. Number of public N. gonorrhoeae genomes in Pathogenwatch by country. [file 13073_2021_858_MOESM1_ESM.pdf]

Table S1. List of studies included in the antimicrobial resistance (AMR) benchmark analyses. The number of isolates included from each study and the antibiotics for which minimum inhibitory concentration (MIC) information is provided is also indicated.

Benchmark (N)	Study	Number of isolates	Project accession	Antibiotics
Test (N=3,987)	Chisholm *et al.* 2015	15	PRJEB14933	Azithromycin, Ceftriaxone
	Golparian *et al.* 2020	183	PRJEB4024	Azithromycin, Ceftriaxone, Cefixime, Ciprofloxacin, Tetracycline, Benzylpenicillin
	Demczuk *et al*. 2015, 2016	382	PRJNA298332<br>PRJNA266539	Azithromycin, Ceftriaxone, Cefixime, Ciprofloxacin, Tetracycline, Benzylpenicillin
	Harris *et al*. 2018	1,054	PRJEB9227	Azithromycin, Ceftriaxone, Cefixime, Ciprofloxacin
	Eyre *et al*. 2017	249	PRJNA315363	Azithromycin, Cefixime, Ciprofloxacin, Tetracycline, Benzylpenicillin
	Fifer *et al*. 2018	101	PRJEB23008	Azithromycin
	Sánchez-Busó *et al*. 2019	403	PRJEB4024	Azithromycin, Ceftriaxone, Cefixime, Ciprofloxacin, Tetracycline, Benzylpenicillin
	Grad *et al*. 2014, 2016	1,114	PRJEB2090<br>PRJEB2999<br>PRJEB7904	Azithromycin, Ceftriaxone, Cefixime, Ciprofloxacin, Tetracycline, Benzylpenicillin
	Jacobsson *et al*. 2016	74	PRJNA322768	Azithromycin, Ceftriaxone, Cefixime, Ciprofloxacin
	Lee *et al*. 2018	398	PRJNA394216	Azithromycin, Ceftriaxone, Cefixime, Ciprofloxacin, Tetracycline, Benzylpenicillin
	Unemo *et al*. 2016	14	PRJEB4024	Azithromycin, Ceftriaxone, Cefixime, Ciprofloxacin, Tetracycline, Benzylpenicillin
Validation (N=1,607)	Town *et al.* 2020	1,288	PRJEB19989	Azithromycin, Ceftriaxone, Cefixime, Ciprofloxacin, Benzylpenicillin
	Yahara *et al.* 2018	245	PRJDB6496<br>PRJDB6504	Azithromycin, Ceftriaxone, Cefixime, Ciprofloxacin
	Kwong *et al*. 2018	75	PRJEB17738	Azithromycin, Ceftriaxone, Ciprofloxacin, Tetracycline, Benzylpenicillin, Spectinomycin

Table S2. Point mutations and genes associated with antimicrobial resistance (AMR) detected by Pathogenwatch on the WHO 2016 reference genome panel. Note that screening for a *porA* mutant gene is included.

Genome Name	Point mutations (SNPs and indels)	Genes
WHO_F	-	-
WHO_G	*folP*_R228S,*gyrA*_S91F,*mtrR*_promoter_a-57del,*parE*_G410V,*penA*_ins346D,*ponA1*_L421P, *rpsJ*_V57M	*tetM*
WHO_K	*folP*_R228S,*gyrA*_D95N,*gyrA*_S91F,*mtrR*_G45D,*mtrR*_promoter_a-57del,*parC*_S87R, *parC*_S88P,*penA*_G545S,*penA*_I312M,*penA*_V316T,*ponA1*_L421P,*porB1b*_A121D, *porB1b*_G120K,*rpsJ*_V57M	-
WHO_L	*gyrA*_D95N,*gyrA*_S91F,*mtrR*_G45D,*mtrR*_promoter_g-131a,*parC*_D86N,*parC*_S88P, *penA*_A501V,*penA*_G542S,*penA*_ins346D,*ponA1*_L421P,*porB1b*_A121D,*porB1b*_G120K, *rpsJ*_V57M	-
WHO_M	*folP*_R228S,*gyrA*_D95G,*gyrA*_S91F,*mtrR*_G45D,*mtrR*_promoter_a-57del,*penA*_ins346D, *ponA1*_L421P,*porB1b*_A121D,*porB1b*_G120K,*rpsJ*_V57M	*bla*TEM
WHO_N	*folP*_R228S,*gyrA*_D95G,*gyrA*_S91F,*mtrR*_A39T,*mtrR*_disrupted,*parC*_S87I,*parE*_G410V, *penA*_ins346D,*ponA1*_L421P,*rpsJ*_V57M	*bla*TEM,*tetM*
WHO_O	16S_rDNA_c1184t,*folP*_R228S,*mtrR*_promoter_a-57del,*penA*_ins346D,*penA*_P551S, *ponA1*_L421P,*porB1b*_A121D,*porB1b*_G120K,*rpsJ*_V57M	*bla*TEM
WHO_P	*folP*_R228S,*mtrR*_disrupted,*penA*_ins346D,*porB1b*_A121D,*rpsJ*_V57M	*mtr*_mosaic_1, *mtrD*_mosaic_1
WHO_U	23S_rDNA_c2597t,*folP*_R228S,*penA*_ins346D,*ponA1*_L421P,*rpsJ*_V57M	*porA*
WHO_V	23S_rDNA_a2045g,*folP*_R228S,*gyrA*_D95G,*gyrA*_S91F,*mtrR*_promoter_a-57del,*parC*_S87R, *penA*_G542S,*penA*_ins346D,*ponA1*_L421P,*porB1b*_A121D,*porB1b*_G120K,*rpsJ*_V57M	*bla*TEM
WHO_W	*folP*_R228S,*gyrA*_D95N,*gyrA*_S91F,*mtrR*_G45D,*mtrR*_promoter_a-57del,*parC*_S87R, *parC*_S88P,*penA*_G545S,*penA*_I312M,*penA*_V316T,*ponA1*_L421P,*porB1b*_A121D, *porB1b*_G120K,*rpsJ*_V57M	-
WHO_X	*folP*_R228S,*gyrA*_D95N,*gyrA*_S91F,*mtrR*_promoter_a-57del,*parC*_S87R,*parC*_S88P, *penA*_A311V,*penA*_G545S,*penA*_I312M,*penA*_T483S,*penA*_V316P,*ponA1*_L421P, *porB1b*_A121D,*porB1b*_G120K,*rpsJ*_V57M	-
WHO_Y	*folP*_R228S,*gyrA*_D95G,*gyrA*_S91F,*mtrR*_promoter_a-57del,*parC*_S87R,*penA*_A501P, *penA*_G545S,*penA*_I312M,*penA*_V316T,*ponA1*_L421P,*porB1b*_A121N,*porB1b*_G120K, *rpsJ*_V57M	-
WHO_Z	*folP*_R228S,*gyrA*_D95N,*gyrA*_S91F,*mtrR*_promoter_a-56c,*parC*_S87R,*parC*_S88P, *penA*_A311V,*penA*_G545S,*penA*_I312M,*penA*_T483S,*penA*_V316T,*ponA1*_L421P, *porB1b*_A121D,*porB1b*_G120K,*rpsJ*_V57M	-

Table S3. Summary of the benchmark analysis of the list of genetic antimicrobial resistance (AMR) mechanisms. AZM = Azithromycin, CIP = Ciprofloxacin, CFM = Cefixime, CRO = Ceftriaxone, PEN = Benzylpenicillin, TET = Tetracycline, SPT = Spectinomycin, TP = True Positives, FP = False Positives, TN = True Negatives, FN = False Negatives, NPV = Negative Predictive Value, CI = Confidence Intervals.

Antibiotic	Dataset	TOTAL	TP	FP	TN	FN	Sensitivity	Specificity	NPV	PPV
AZM	Test<br>(CI)	3679	468	17	3008	186	71.56<br>(67.93-74.99)	99.44<br>(99.10-99.67)	94.18<br>(93.31-94.96)	96.49<br>(94.45-97.95)
	Validation<br>(CI)	1573	8	7	1556	2	80.00<br>(44.39-97.48)	99.55<br>(99.08-99.82)	99.87<br>(99.54-99.98)	53.33<br>(26.59-78.73)
CFM	Test<br>(CI)	3601	371	323	2892	15	96.11<br>(93.67-97.81)	89.95<br>(88.86-90.97)	99.48<br>(99.15-99.71)	53.46<br>(49.67-57.22)
	Validation<br>(CI)	1498	68	137	1289	4	94.44<br>(86.38-98.47)	90.39<br>(88.74-91.87)	99.69<br>(99.21-99.92)	33.17<br>(26.77-40.07)
CIP	Test<br>(CI)	3281	1548	15	1671	47	97.05<br>(96.10-97.83)	99.11<br>(98.54-99.50)	97.26<br>(96.38-97.98)	99.04<br>(98.42-99.46)
	Validation<br>(CI)	1290	549	4	693	44	92.58<br>(90.17-94.56)	99.43<br>(98.54-99.84)	94.03<br>(92.07-95.63)	99.28<br>(98.16-99.80)
CRO	Test<br>(CI)	3635	9	5	3603	18	33.33<br>(16.52-53.96)	99.86<br>(99.68-99.95)	99.50<br>(99.22-99.71)	64.29<br>(35.14-87.24)
	Validation<br>(CI)	1571	3	0	1565	3	50.00<br>(11.81-88.19)	100.00<br>(99.76-100)	99.81<br>(99.44-99.96)	100.00<br>(29.24-100)
PEN	Test<br>(CI)	1654	1424	46	157	27	98.14<br>(97.30-98.77)	77.34<br>(70.96-82.91)	85.33<br>(79.37-90.10)	96.87<br>(95.85-97.70)
	Validation<br>(CI)	1330	1228	86	7	9	99.27<br>(98.62-99.67)	7.53<br>(3.08-14.90)	43.75<br>(19.75-70.12)	93.46<br>(91.98-94.73)
TET	Test<br>(CI)	1661	1096	215	341	9	99.19<br>(98.46-99.63)	61.33<br>(57.14-65.40)	97.43<br>(95.17-98.82)	83.60<br>(81.48-85.57)
	Validation<br>(CI)	75	74	0	0	1	98.67<br>(92.79-99.97)	-	0.00<br>(0.00-97.50)	100.00<br>(95.14-100)
SPT	Validation<br>(CI)	75	0	0	75	0	-	100.00<br>(95.20-100)	100.00<br>(95.20-100)	-

Table S4. List of genetic mechanisms detected on the test benchmark (N=3,987) for each of the six main antibiotics. The total number of isolates carrying each mechanism and the benchmark results are shown. AZM = Azithromycin, CIP = Ciprofloxacin, CFM = Cefixime, CRO = Ceftriaxone, PEN = Benzylpenicillin, TET = Tetracycline, SPT = Spectinomycin, TP = True Positives, FP = False Positives, PPV = Positive Predictive Values.

Agent	Mechanism	Total	TP	FP	PPV
AZM	23S_rDNA_a2045g	108	106	2	98.15
AZM	23S_rDNA_c2597t	340	331	9	97.35
AZM	23S_rDNA_c2597t__*mtrR*_promoter_a-57del__*mtrR*_G45D	6	6	0	100
AZM	*ermB*	1	1	0	100
AZM	*ermC*	2	2	0	100
AZM	*mtr*_mosaic_1	6	4	2	66.67
AZM	*mtr*_mosaic_2	24	21	3	87.5
AZM	*mtr*_mosaic_3	1	1	0	100
AZM	*mtrD*_mosaic_1	8	5	3	62.5
AZM	*mtrD*_mosaic_2	21	19	2	90.48
AZM	*mtrD*_mosaic_3	1	1	0	100
AZM	*rplD*_G70D__23S_rDNA_c2597t	5	5	0	100
AZM	*rplV*_------83KGPSLK	1	1	0	100
AZM	*rplV*_----90ARAK	1	1	0	100
CFM	*penA*_A311V_G545S_I312M_T483S_V316P	1	1	0	100
CFM	*penA*_A311V_G545S_I312M_T483S_V316T	1	1	0	100
CFM	*penA*_A501P	4	4	0	100
CFM	*penA*_G545S_I312M_V316T	690	367	323	53.19
CFM	*penA*_G545S_I312M_V316T__*mtrR*_G45D	50	39	11	78
CFM	*penA*_T483S	2	2	0	100
CFM	*penA*_V316P	1	1	0	100
CFM	*rpoB*_R201H	1	1	0	100
CFM	*rpoB*_R201H__*mtrR*_G45D	1	1	0	100
CFM	*rpoD*_A95-_D92-_D93-_D94-	1	1	0	100
CFM	*rpoD*_A95-_D92-_D93-_D94-__*mtrR*_G45D	1	1	0	100
CFM	*rpoD*_E98K	1	1	0	100
CFM	*rpoD*_E98K__*mtrR*_G45D	1	1	0	100
CIP	*gyrA*_D95A	227	225	2	99.12
CIP	*gyrA*_D95G	1199	1192	7	99.42
CIP	*gyrA*_D95N	57	57	0	100
CIP	*gyrA*_S91F	1511	1503	8	99.47
CIP	*parC*_D86N	90	90	0	100
CIP	*parC*_E91K	22	22	0	100
CIP	*parC*_S87I	14	14	0	100
CIP	*parC*_S87N	98	98	0	100
CIP	*parC*_S87R	1050	1037	13	98.76
CIP	*parC*_S88P	25	25	0	100
CIP	*parE*_G410V	5	5	0	100
CRO	*penA*_A311V_G545S_I312M_T483S_V316P	1	1	0	100
CRO	*penA*_A311V_G545S_I312M_T483S_V316T	1	1	0	100
CRO	*penA*_A501P	4	4	0	100
CRO	*penA*_A501V_G542S	5	2	3	40
CRO	*penA*_T483S	2	2	0	100
CRO	*penA*_V316P	1	1	0	100
CRO	*rpoB*_R201H	1	1	0	100
CRO	*rpoD*_A95-_D92-_D93-_D94-	1	0	1	0
CRO	*rpoD*_E98K	1	0	1	0
PEN	*blaTEM*	133	129	4	96.99
PEN	*mtrR*_A39T__*porB1b*_A121D_G120K	31	31	0	100
PEN	*mtrR*_disrupted	67	65	2	97.01
PEN	*mtrR*_G45D	231	218	13	94.37
PEN	*mtrR*_promoter_a-56c	1	1	0	100
PEN	*mtrR*_promoter_a-57del	687	665	22	96.8
PEN	*mtrR*_promoter_a-57del__*penA*_G542S__*porB1b*_A121D_G120K	42	41	1	97.62
PEN	*mtrR*_promoter_a-57del__*porB1b*_A121N_G120K	286	276	10	96.5
PEN	*mtrR*_promoter_g-131a	9	9	0	100
PEN	*penA*_A501P	4	4	0	100
PEN	*penA*_A501T	55	55	0	100
PEN	*penA*_A501V	30	30	0	100
PEN	*penA*_G542S	120	118	2	98.33
PEN	*penA*_G545S	297	289	8	97.31
PEN	*penA*_G545S_I312M_V316T__*porB1b*_A121D_G120K	17	17	0	100
PEN	*penA*_I312M	316	306	10	96.84
PEN	*penA*_ins346D	1124	1091	33	97.06
PEN	*penA*_P551S	92	87	5	94.57
PEN	*penA*_T483S	2	2	0	100
PEN	*penA*_V316P	1	1	0	100
PEN	*penA*_V316T	315	305	10	96.83

PEN	*ponA1*_L421P	786	764	22	97.2
PEN	*porB1b*_A121D	323	314	9	97.21
PEN	*porB1b*_A121D__*ponA1*_L421P__*mtrR*_G45D__*mtrR*_promoter_a-57del	34	33	1	97.06
PEN	*porB1b*_A121N	305	293	12	96.07
PEN	*porB1b*_A121N_G120K__*ponA1*_L421P__*mtrR*_promoter_a-57del	269	259	10	96.28
PEN	*porB1b*_G120K	618	597	21	96.6
TET	*mtrR*_disrupted	67	62	5	92.54
TET	*mtrR*_promoter_a-56c	1	1	0	100
TET	*mtrR*_promoter_a-57del	691	651	40	94.21
TET	*mtrR*_promoter_a-57del__*rpsJ*_V57M	681	646	35	94.86
TET	*mtrR*_promoter_a-57del__*rpsJ*_V57M__*mtrR*_G45D	83	78	5	93.98
TET	*mtrR*_promoter_g-131a	9	7	2	77.78
TET	*rpsJ*_V57M	1292	1083	209	83.82
TET	*rpsJ*_V57M__*mtrR*_A39T__*mtrR*_promoter_a-57del	6	5	1	83.33
TET	*rpsJ*_V57M__*mtrR*_A39T_disrupted	51	51	0	100
TET	*rpsJ*_V57M__*mtrR*_A39T_G45D	3	3	0	100
TET	*rpsJ*_V57M__*mtrR*_promoter_a-56c	1	1	0	100
TET	*tetM*	245	243	2	99.18

Table S5. List of genetic mechanisms detected on the validation benchmark (N=1,607) for each of the six main antibiotics. The total number of isolates carrying each mechanism and the benchmark results are shown. AZM = Azithromycin, CIP = Ciprofloxacin, CFM = Cefixime, CRO = Ceftriaxone, PEN = Benzylpenicillin, TET = Tetracycline, SPT = Spectinomycin, TP = True Positives, FP = False Positives, PPV = Positive Predictive Values.

Agent	Mechanism	Total	TP	FP	PPV
AZM	23S_rDNA_a2045g	3	3	0	100
AZM	23S_rDNA_c2597t	3	3	0	100
AZM	*mtr*_mosaic_1	2	1	1	50
AZM	*mtr*_mosaic_2	8	2	6	25
AZM	*mtrD*_mosaic_1	1	1	0	100
AZM	*mtrD*_mosaic_2	6	2	4	33.33
CFM	*penA*_A311V_G545S_I312M_T483S_V316T	3	3	0	100
CFM	*penA*_G545S_I312M_V316T	205	68	137	33.17
CFM	*penA*_G545S_I312M_V316T__*mtrR*_G45D	32	21	11	65.63
CFM	*penA*_T483S	3	3	0	100
CIP	*gyrA*_D95A	188	185	3	98.4
CIP	*gyrA*_D95G	309	308	1	99.68
CIP	*gyrA*_D95N	55	55	0	100
CIP	*gyrA*_S91F	549	545	4	99.27
CIP	*parC*_D86N	90	90	0	100
CIP	*parC*_E91K	4	4	0	100
CIP	*parC*_S87I	6	6	0	100
CIP	*parC*_S87N	31	30	1	96.77
CIP	*parC*_S87R	321	320	1	99.69
CIP	*parC*_S88P	44	44	0	100
CRO	*penA*_A311V_G545S_I312M_T483S_V316T	3	3	0	100
CRO	*penA*_T483S	3	3	0	100
PEN	*blaTEM*	561	525	36	93.58
PEN	*mtrR*_A39T__*porB1b*_A121D_G120K	11	11	0	100
PEN	*mtrR*_disrupted	94	91	3	96.81
PEN	*mtrR*_G45D	144	141	3	97.92
PEN	*mtrR*_promoter_a-56c	2	2	0	100
PEN	*mtrR*_promoter_a-57del	395	388	7	98.23
PEN	*mtrR*_promoter_a-57del__*penA*_G542S__*porB1b*_A121D_G120K	2	2	0	100
PEN	*mtrR*_promoter_a-57del__*porB1b*_A121N_G120K	60	60	0	100
PEN	*mtrR*_promoter_g-131a	3	3	0	100
PEN	*penA*_A501T	98	98	0	100
PEN	*penA*_A501V	30	30	0	100
PEN	*penA*_G542S	31	31	0	100
PEN	*penA*_G545S	93	93	0	100
PEN	*penA*_G545S_I312M_V316T__*porB1b*_A121D_G120K	2	2	0	100
PEN	*penA*_I312M	94	94	0	100
PEN	*penA*_ins346D	1208	1125	83	93.13
PEN	*penA*_P551S	42	42	0	100
PEN	*penA*_V316T	94	94	0	100
PEN	*ponA1*_L421P	464	455	9	98.06
PEN	*porB1b*_A121D	192	189	3	98.44
PEN	*porB1b*_A121D__*ponA1*_L421P__*mtrR*_G45D__*mtrR*_promoter_a-57del	11	10	1	90.91
PEN	*porB1b*_A121N	83	83	0	100
PEN	*porB1b*_A121N_G120K__*ponA1*_L421P__*mtrR*_promoter_a-57del	60	60	0	100
PEN	*porB1b*_G120K	283	281	2	99.29
TET	*mtrR*_disrupted	2	2	0	100
TET	*mtrR*_promoter_a-57del	37	37	0	100
TET	*mtrR*_promoter_a-57del__*rpsJ*_V57M	37	37	0	100
TET	*mtrR*_promoter_a-57del__*rpsJ*_V57M__*mtrR*_G45D	3	3	0	100
TET	*mtrR*_promoter_g-131a	3	3	0	100
TET	*rpsJ*_V57M	74	74	0	100
TET	*rpsJ*_V57M__*mtrR*_A39T_disrupted	2	2	0	100
TET	*tetM*	5	5	0	100

Table S6. Published studies on *N. gonorrhoeae* genomics for which new public collections have been created in Pathogenwatch sorted by included number of isolates. Dates correspond to those in the final collections.

PubMed ID	ENA project accession(s)	Collection label	Number of isolates in publication	Number of isolates in collection	Geographical location and timescale
31488838	PRJNA520805	Williamson *et al*, 2019	2,186	2,179	Australia<br>2017
27427203	PRJNA315363	De Silva *et al*, 2016	1,872	1,783	Brighton, United Kingdom (UK)<br>2004-2015
31978353,<br>32091356	PRJEB19989	Town *et al*, 2020*	1,277	1,288	England, UK<br>2013-2016
27638945	PRJEB2090<br>PRJEB2999<br>PRJEB7904	Grad *et al*, 2016*	1,102	1,035	United States (US)<br>2000-2013
32829411	PRJEB10016	Mortimer *et al*, 2020	897	891	New York City, US<br>2011-2015
32213251	PRJEB32435	Alfsnes *et al*, 2020	958	816	Norway<br>2016-2017
30788502	PRJNA317462<br>PRJNA329501	Thomas *et al*, 2019	649	644	United States<br>2014-2016
31358980	PRJEB4024	Sánchez-Busó *et al*, 2019	419	395	Worldwide<br>1979-2013
29182725	PRJNA394216	Lee *et al*, 2018	398	376	New Zealand<br>2014-2015
32071056	PRJNA317462<br>PRJNA329501	Schmerer *et al*, 2020	334	324	United States<br>2016
30063202	PRJDB6496<br>PRJDB6504	Yahara *et al*, 2018	271	245	Kyoto and Osaka, Japan<br>2011-2015
32068837	PRJEB34425	Lan *et al*, 2020	229	227	Vietnam<br>2011-2016
24462211	PRJEB2090<br>PRJEB2999<br>PRJEB7904	Grad *et al*, 2014*	236	216	United States<br>2009-2010
26935729	PRJNA298332	Demczuk *et al*, 2016	246	200	Canada<br>1997-2014
27353752	PRJEB2124	Didelot *et al*, 2016	237	194	Sheffield and London, UK<br>1995-2004
32013864	PRJEB4024	Golparian *et al*, 2020	231	192	Denmark<br>1928-2013
25378573	PRJNA266539	Demczuk *et al*, 2015	180	168	Canada<br>1989-2013
33200978	PRJEB32435	Osnes *et al*, 2020	148	133	Norway<br>2015-2018
29701830	PRJEB10104	Cehovin *et al*, 2018*	103	112	Coastal Kenya<br>2010-2015
29523496	PRJEB23008	Fifer *et al*, 2018	101	100	England, UK<br>2004-2017
29367612	PRJNA392203	Buckley *et al*, 2018	94	92	Australia<br>2012-2014
29247013	PRJEB17738	Kwong *et al*, 2018	94	75	Australia<br>2006-2014

28348871	PRJEB14168	Kwong *et al*, 2016;<br>Martin et al, 2004	50	48	Australia and New Zealand<br>2004-2015
29882175	PRJNA473385	Ryan *et al*, 2018	43	42	Ireland<br>2012-2016
28510723	PRJNA348107	Wind *et al*, 2017	31	23	Amsterdam, Netherlands<br>2002-2012
25780762	**	Ezewudo *et al*, 2015	61	18	Worldwide<br>1982-2008
26601852	PRJEB14933	Chisholm *et al*, 2015	15	14	Leeds, UK<br>2015

* In these collections, the number of accession numbers on the corresponding ENA project was higher than the number in the final publication. These genomes were included in the corresponding collections.
** Multiple ENA project accessions are linked to Ezewudo *et al*, 2015: PRJNA209340, PRJNA209307, PRJNA209319, PRJNA209352, PRJNA209376, PRJNA209466, PRJNA209373, PRJNA209465, PRJNA209320, PRJNA209333, PRJNA209345, PRJNA209351, PRJNA209342, PRJNA209343, PRJNA209347, PRJNA209470, PRJNA244850, PRJNA209316.

Table S7. Number of public *N. gonorrhoeae* genomes in Pathogenwatch clustered by country.

Country	Number of isolates	Individual proportion (%)	Cumulative sum	Cumulative proportion (%)
United Kingdom	3476	27.77	3476	27.77
United States	2774	22.17	6250	49.94
Australia	2388	19.08	8638	69.02
Norway	990	7.91	9628	76.93
New Zealand	396	3.16	10024	80.10
Canada	382	3.05	10406	83.15
Japan	268	2.14	10674	85.29
Denmark	255	2.04	10929	87.33
Vietnam	233	1.86	11162	89.19
Spain	129	1.03	11291	90.22
Kenya	112	0.89	11403	91.11
Portugal	108	0.86	11511	91.98
Netherlands	92	0.74	11603	92.71
Slovenia	77	0.62	11680	93.33
France	62	0.50	11742	93.82
Belgium	55	0.44	11797	94.26
Austria	54	0.43	11851	94.69
Greece	54	0.43	11905	95.13
Germany	53	0.42	11958	95.55
Sweden	51	0.41	12009	95.96
Hungary	48	0.38	12057	96.34
Ireland	42	0.34	12099	96.68
Slovakia	39	0.31	12138	96.99
Latvia	38	0.30	12176	97.29
Poland	34	0.27	12210	97.56
Scotland	30	0.24	12240	97.80
Italy	28	0.22	12268	98.03
Belarus	24	0.19	12292	98.22
India	24	0.19	12316	98.41
Guinea-Bissau	22	0.18	12338	98.59
Thailand	22	0.18	12360	98.76
Malta	20	0.16	12380	98.92
Estonia	17	0.14	12397	99.06
Pakistan	14	0.11	12411	99.17
Philippines	14	0.11	12425	99.28
Russia	13	0.10	12438	99.38
Cyprus	8	0.06	12446	99.45
Bhutan	7	0.06	12453	99.50
China	7	0.06	12460	99.56
Gambia	5	0.04	12465	99.60
Iceland	5	0.04	12470	99.64
Indonesia	5	0.04	12475	99.68
Cuba	4	0.03	12479	99.71
Turkey	4	0.03	12483	99.74
Morocco	3	0.02	12486	99.77
South Africa	3	0.02	12489	99.79
Brasil	2	0.02	12491	99.81
Cabo Verde	2	0.02	12493	99.82
Chile	2	0.02	12495	99.84
Ivory Coast	2	0.02	12497	99.86
Suriname	2	0.02	12499	99.87
Tanzania	2	0.02	12501	99.89
Angola	1	0.01	12502	99.90
Argentina	1	0.01	12503	99.90
Armenia	1	0.01	12504	99.91
Bulgaria	1	0.01	12505	99.92
Caribbean	1	0.01	12506	99.93
Ecuador	1	0.01	12507	99.94
Finland	1	0.01	12508	99.94
Guinea	1	0.01	12509	99.95
Jamaica	1	0.01	12510	99.96
Lithuania	1	0.01	12511	99.97
Malaysia	1	0.01	12512	99.98
Romania	1	0.01	12513	99.98
Saudi Arabia	1	0.01	12514	99.99
Uganda	1	0.01	12515	100.00
